# The evolution of cleavage voting in four Western countries: Structural, behavioural or political dealignment?

**DOI:** 10.1111/1475-6765.12336

**Published:** 2019-03-29

**Authors:** ANDREAS C. GOLDBERG

**Affiliations:** ^1^ University of Amsterdam (ASCoR) The Netherlands

**Keywords:** voting behaviour, turnout, cleavages, religion, social class

## Abstract

Since the heyday of cleavage voting in the 1960s and 1970s, the majority of studies presents evidence of a decline in cleavage voting – caused by either structural or behavioural dealignment. Structural dealignment denotes changes in group size responsible for a decrease in cleavage voting, whereas behavioural dealignment concerns weakening party–voter links over time. A third phenomenon posited in this article is the collective voting abstention of certain (social) groups, here referred to as ‘political dealignment’, which results in a new type of division of voting versus abstention. The purpose of this article is to examine the three underlying mechanisms for the decline in social class and religious cleavage voting across four Western countries (Great Britain, the Netherlands, Switzerland and the United States) over the last 40–60 years using longitudinal post‐election data. The results prove a strong presence of political dealignment and increasing turnout gaps regarding both the class and religious cleavage. Furthermore, whenever a decline in cleavage voting is present, it is mainly caused by changes in the social groups’ behaviour and less by changing social structures in a country.

## Introduction

Fifty years ago, cleavages such as social class and religion were important determinants of voting behaviour. Since that heyday, several scholars have reported a decline in influence of cleavages (e.g., Dalton 2002; Franklin et al. 1992), fuelled by a broad range of factors such as societal modernisation, globalisation, cognitive mobilisation and increasing media coverage. The resulting individualisation of politics has caused a shift in electoral decision making. Instead of relying on sociostructural reference groups and party cues, citizens now have the capability and knowledge to decide which party to vote for based on policy preferences, issue interests or performance judgments (Dalton 1998).

Traditional approaches that examine the decreasing relevance of party voting by cleavage groups distinguish between structural and/or behavioural effects. ‘Structural dealignment’ refers to changes in group size, such as a declining group of workers or religious people might be responsible for a decrease in cleavage voting. ‘Behavioural dealignment’, by contrast, concerns weakening party‐voter links, like those between workers and left parties. This article adds a relatively contemporary third phenomenon to the existing two approaches: collective voting abstention by certain social groups (e.g., the lower classes), here referred to as ‘political dealignment’. Nowadays, belonging to a cleavage group may express itself not so much in a certain party vote, but increasingly in a new division that differentiates citizens by whether or not they have voted (cf. Evans 2000).

The vast amount of cleavage studies attests these broad developments. However, the majority of studies do not distinguish between different mechanisms (exceptions are Lachat 2007a; Best 2011; Elff & Roßteutscher 2017; Goldberg 2017; Heath 2018), and even those few that do suffer from the same limitations as many other cleavage studies in that they focus on only one specific country (e.g., De Graaf et al. 2001; Evans & Tilley 2012; Elff & Roßteutscher 2017; Goldberg 2017; Heath 2018); consider only one particular cleavage or cleavage group, such as workers (e.g., Clark et al. 1993; Raymond 2011; Jansen et al. 2013; Goldberg 2014; Heath 2018); or only analyse specific parties or merged (left versus right) party blocs (e.g., Franklin et al. 1992; Elff 2007; Best 2011; Jansen et al. 2013; Elff & Roßteutscher 2017). The aim of this article is to offer a more comprehensive analysis of all three underlying mechanisms of (declining) cleavage voting. Focusing on the religious and social class cleavages, the analysis relies on longitudinal post‐election data across four Western countries, which differ in the timing of the cleavage decline: Great Britain, the Netherlands, Switzerland and the United States. The use of the so‐called ‘lambda index’ (Lachat 2007b) as an analytical tool provides a comparable measure of cleavage strength across time, but more importantly allows for a separation of the effects due to structural and behavioural changes. In addition, the article is among the first to link the traditional analysis of cleavage voting focusing on party choice with growing differences in turnout.

The article contributes to the extant literature in three ways. First, it adds to the few studies that jointly analyse the aforementioned aspects of the development of cleavage voting and hence allows for separating out the underlying mechanisms. As a starting point, testing these mechanisms with recent data from the four countries updates existing findings and contributes to the partly still ongoing discussion about the extent of declining cleavage voting (e.g., Brooks et al. 2006; Elff 2007; Raymond 2011). Second, as opposed to merging variables into simplified party and cleavage groups, the analysis relies on detailed party choice and cleavage measures that represent empirical complexity. Furthermore, these variables are examined using the same statistical models across countries. This detailed but still comparable approach allows for a generalisation of the developments. A final, third, contribution is the joined analysis of party choice and turnout in the context of cleavage theory. Although there are strong theoretical links between turnout and party choice, few studies examine both electoral aspects at the same time (for notable exceptions, see Hout et al. 1995; Rennwald 2014; Elff & Roßteutscher 2017; Goldberg 2017; Heath 2018).

## Theory

The literature offers various definitions of cleavage and methods for measuring its impact (e.g., Bartolini & Mair 1990). The ‘impact’ itself, though, is rarely conceptualised in detail, despite being crucial when examining cleavage evolution over time. Brooks et al.’s (2006: 92) study is an exception by explicitly offering such a definition: cleavage impact is understood ‘in terms of the magnitude of the average difference in political alignment among groups comprising a particular cleavage’. Crucial to this definition is the comparison of the voting behaviour of one group with the behaviour of at least one other group. Elff and Rossteutscher (2011) argue that cleavage voting is often wrongly interpreted as strong voting by one social group for ‘their’ party, such as workers voting for social democrats. Such behaviour only leads to a cleavage impact when other social groups differ in their voting behaviour and when the respective cleavage groups comprise a sufficiently large number of members (Mair 2008; Best 2011). Furthermore, the general increase in abstention over recent decades indicates that voting behaviour today also includes the active decision to not vote for any party. Cleavage variables may thus be important determinants not only for party choice, but also for the decision to abstain.

The concept of cleavage impact thus focuses both on the behaviour of whole social groups in relation to each other – looking at party choice and turnout – and examines the groups’ sizes. The ensuing sections in this article discuss the relevance of the different elements and potential changes over time in more depth with a focus on abstention as a new element of cleavage voting.

## Dealignment in the context of party voting

In the context of declining cleavage party voting, the approach of dealignment stands for a weakening relationship between social characteristics and voting behaviour. This development may be caused by two different processes. The first process assumes that formerly strong alliances between social groups and political parties have softened. The underlying change in partisan alignment of certain groups is referred to as ‘behavioural dealignment’ (Lachat 2007a: 68).^1^ A less homogeneous party vote among social groups then results in the weakening of the corresponding cleavage. The second effect is a change in the relative size of the social groups. This phenomenon is called ‘structural dealignment’ (Bornschier & Helbling 2005: 29; Brooks et al. 2006: 91; Lachat 2007a: 68). The importance of a cleavage has weakened simply because of a reduction or even ‘extinction’ of a social group, and not because the linking issue or subject has lost effect on vote choice.

Best (2011) distinguishes between the electoral relevance of a cleavage group in terms of size and that group's voting behaviour. Whereas evidence of declining group sizes of formerly influential social groups points towards a decline in electoral relevance of these groups, Evans (2010: 643) argues that a shrinking group might be more distinctive in its voting behaviour compared to a larger one. Moreover, and in contrast to the possibility that some groups may (again) change their behaviour in the future, a decline in size is more or less irreversible (Best 2011). Thus, scholars like Mair (2008) acknowledge that the changing group sizes of traditional cleavage groups contribute to the general trend of declining cleavage voting. Other scholars such as Elff and Rossteutscher (2011) disagree, claiming that, strictly speaking, a numerical decline is less relevant for analysing social cleavages. Since the cleavage model highlights the ‘consequences of social *divisions* on voting’ (Elff & Rossteutscher 2011: 109; emphasis in the original), the size of a social group does not have a direct effect on the impact of these divisions. In compliance with this view, most studies examine behavioural change only and neglect the potential importance of changes in group size. A reduction in group size, though, becomes very relevant from a party perspective, since electoral support for an increasingly shrinking social group might not be enough for parties to successfully compete in the electoral arena. Consequently, parties may adapt their strategies to mobilise voters from different social groups, which in turn may add to behavioural dealignment (by their traditional electorate), as mentioned earlier.

One prominent reason for the decline in cleavage voting assumes that rising levels of economic security have led to a weakening of those material values that used to be prevalent among older generations (Inglehart 1977; Van Deth 1995; Inglehart & Baker 2000; Elff 2007). At the same time, other developments, such as the expansion of the welfare state, the diversification of mass media, secularisation and rising levels of education, have created a stronger emphasis on social and cultural issues. Especially due to improved education levels, known under the concept of ‘cognitive mobilisation’, traditional sources of information like labour unions or the church have become less important, leading to voting choices independent of belonging to a certain social group (Dalton 1984; Manza & Brooks 1999; Enyedi 2008). Other developments such as tertiarisation, social and geographical mobility, growing multiculturalism and the increasing complexity of modern issues like globalisation and international terrorism simply do not fit into traditional patterns of party competition related to cleavages (Norris & Inglehart 2004; Oesch & Rennwald 2010).^2^ These aspects have resulted in the increasing heterogeneity of individual life and the fragmentation of the social structure (Kriesi 2010). This translates into political life as an ‘individualisation of politics’ (Dalton 1998: 346; Thomassen 2005: 16). A sense of solidarity among parts of the electorate becomes difficult to identify; people behave as isolated individuals instead of members of a (shrinking) social group or community (Dogan 1995; Kriesi 2010).

The first two hypotheses represent expectations commonly reported in the literature, although conflicting findings can (still) be found. The novel examination of traditional cleavage voting should thus help to reach an agreement about the extent of, and reasons for, the decline – and also allows us to directly link this decline to more recent developments regarding turnout. The hypotheses assume a declining impact of cleavages on party choice due to both behavioural and structural dealignment:

*H1*: Cleavage voting has declined due to behavioural changes.
*H2*: Cleavage voting has declined due to structural changes.

Today, an analysis of cleavage voting in terms of party choice is no longer sufficient. In the context of class voting, Verba et al. (1978) argue that the institutionalisation of cleavages in the political system prevents class differences in turnout. However, as soon as the party system fails to represent all (relevant) class interests, the non‐represented classes may react with abstention, which leads to a general class bias in turnout. Resultantly, class differences today may not (or they may less) express themselves in a certain party vote, but are rather seen in a new class‐vote division that differentiates citizens by whether they have voted or not (cf. Evans 2000; Evans & Tilley 2017; Goldberg 2017). The next section discusses the class‐vote division in more detail and extends the argument to the religious cleavage as well.

## Abstention as a new form of dealignment?

Similar to Verba et al. (1978), Weakliem and Heath (1999) argue that one has to consider the level of non‐voting in the context of class voting (cf. Elff & Roßteutscher 2017; Heath 2018), particularly as a significant part of the eligible population does not vote. This argument makes sense since, in general, turnout is lower among the working class compared to the middle class. This line of argument comes from the United States, where research suggests an unusually small class influence on party choice, but a particularly strong influence on electoral participation (e.g., Manza & Brooks 1999; Kerbo & Gonzalez 2003). The explanation for this pattern, however, is not restricted to the United States. The argument suggests that changing party strategies (e.g., the decreasing representation of the working class by social democrat parties) may lead to non‐voting among certain social groups (see also Verba et al. 1978). An alternative explanation states that people from the lower classes possess less political knowledge and interest, and thus consider voting as less of an obligation of citizenship (cf. Weakliem & Heath 1999).

Both explanations also appear in Brady et al.’s (1995: 271) three arguments for non‐participation in politics, that citizens abstain ‘because they can't, because they don't want to, or because nobody asked’. Such arguments are not specific to the class cleavage, but hold equally for religion. The first reason, that *they can't*, refers to an unequal distribution of resources, such as civic skills, between groups. For the class cleavage, occupation and resultant job opportunities determine the level of civic skills, whereas for religion this level is determined by (active) involvement in the church (Brady et al. 1995). The idea that *they don't want to* refers to an absence of psychological engagement – for example, people feel less of a civic duty and/or obligation to vote. Finally, that *nobody asked* highlights the different mobilisation strategies of parties. In contrast to the two ‘bottom‐up’ factors, this third factor emphasises the crucial role of parties and the related ‘top‐down’ perspective. Although party strategies are at least partly shaped by structural changes themselves (Kitschelt 1994; Jansen et al. 2013), some authors highlight specifically the top‐down approach for group‐specific abstention (e.g., Verba et al. 1978; Heath 2018).

Overall, people may have different reasons for switching from voting to abstention, what I term ‘political dealignment’. On the one hand, growing inequality in the distribution of resources over recent decades (e.g., Schäfer 2010) and the declining relevance of labour unions or churches may result in a more skewed distribution of civic skills and ultimately in abstention for groups with fewer civic skills (cf. Blais 2000) (*bottom‐up*). On the other hand, party positions and related strategies also adapt over time (e.g., Elff 2009; Evans & De Graaf 2013; Jansen et al. 2013; Evans & Tilley 2017; Rennwald & Evans 2014), in the sense that especially shrinking groups such as workers or religious people may today be less actively mobilised/targeted by parties compared to some decades ago (*top‐down*).

Empirically, one can observe a general decrease in turnout in most countries. However, it is indeed mostly the least‐well off and lower classes that tend to abstain more often (e.g., Leighley & Nagler 1992). Hence, a growing turnout gap is especially likely to be found between less well‐off classes like workers and more affluent classes such as managers. The third hypothesis thus states:

*H3*: The decrease in electoral participation has been stronger among less well‐off classes than among more affluent classes.

In a similar way to class cleavage, unequal participation is also possible in the religious cleavage. First, today's religious party offer is less clear‐cut than before. Following the process of secularisation and the decreasing size of specific religious groups (e.g., active churchgoers or particular denominations) religious parties have opened up to a broader electorate – and by doing so, have weakened their religious profile. This effect should be especially relevant in multiparty systems with (single‐issue) parties that strongly or even exclusively represent religious issues. One example would be the CVP (Christian Democrats) in Switzerland, which has traditionally represented only Catholics, but in recent decades opened up to Christians in general. However, this new profile has not attracted many Protestants, but instead led to a further decline in Catholic supporters. A similar weakening of the profile may happen after party mergers. Such an example is the CDA (Christian Democratic Appeal) in the Netherlands, which evolved out of three smaller parties representing Catholics, Protestants and Calvinists. Today, all three denominational groups are less represented with their distinct values in the merged party (De Graaf et al. 2001). Both changes on the party stage may result in a feeling among religious people that their values and opinions are less represented, which eventually may lead them to abstain (*top‐down*).

However, as the studies in Engeli et al. (2012) show, the non‐party‐specific and more general importance of religion on the political stage – especially in terms of morality issues such as abortion, same‐sex marriage, euthanasia or stem‐cell research – differs strongly between countries. Whereas Switzerland and the Netherlands traditionally belong to the ‘religious world’ with a high relevance of morality issues, the United Kingdom is the opposite – belonging to the ‘secular world’. The United States joined the religious world in recent decades, which rather speaks for the enduring participation of religious people (see also Wielhouwer 2009). A generally opposing argument to a declining turnout among religious people is the (still) stronger civic norms of these people (Blais 2000), leading them to resist the general trend of disenchantment with politics more strongly (e.g., Elff & Roßteutscher 2017), and this even more so when morality issues are prominent on the political stage. In that case, religious people continue to have attributes that *they can* and that *they want to* participate politically (*bottom‐up*).

In sum, changes on the party stage, including an opening up of parties to a wider electorate and party mergers, may lead religious people to abstain. By contrast, the ongoing political relevance of religion regarding morality issues and the still higher civic norms among religious people suggest a more stable participation for religious persons in comparison to non‐religious people, who may follow the general trend of abstention more strongly. The last hypothesis follows this second line of reasoning and expects a (further) growing turnout gap following the weaker political dealignment among religious people:

*H4*: The decrease in electoral participation has been weaker among religious people than among non‐religious people.

## Data and method

The analysis is based on four Western countries: Great Britain, the Netherlands, Switzerland and the United States. Both theoretical and empirical factors motivate this selection. A first important commonality of the chosen countries is their Western culture and long‐term democratic tradition. Further, religious and class cleavages have been important in all four political systems in the past, albeit with varying strengths. A final similarity is that all four countries witnessed a decline in cleavage voting over the last decades, though beginning at various points in time (e.g., Brooks et al. 2006; Franklin et al. 1992). The latter difference is crucial as the timing of the cleavage decline may matter for the influence and pattern of the three forms of dealignment – for example, a turnout gap may open only after cleavage voting lost its relevance. For that reason, the four countries represent a variety of a historical (United States), an early (Great Britain), a middle (the Netherland) and rather late decline (Switzerland) country (see Franklin et al. 1992). This ensures we can detect possible relationships between the different forms of dealignment – that is, regarding party choice and turnout. A more practical reason for the country choice is data availability over a long period of time. Data stem from the national election studies, which offer detailed information about class and religion for the last 40–60 years (Great Britain 1964–2015; the Netherlands 1971–2012; Switzerland 1971–2015; United States 1952–2012). A list of the separate datasets can be found online in Appendix Table 1. The alternative usage of existing comparative datasets including (presumably) similar variable codings across countries and across time implies a loss of detailed information due to merging and simplification of country‐specific codings (e.g., merging parties into (left versus right) party blocs or the harmonisation of country‐specific class schemes).^3^ In contrast, the use of separate national datasets allows for a detailed and adequate coding of the cleavages in each country. Still, the aim is to have similar measures across countries, which, however, in the case of social class is not always possible. The focus on four countries allows for a necessary detailed discussion, but at the same time provides some comparison resulting in more general findings beyond country‐specific developments.

## Operationalisation

### Dependent variables

Two aspects of electoral behaviour are important for the following analysis: differences in party voting, and differences in electoral participation. Both are measured at the individual level. In terms of the first dependent variable, the respective questions ask respondents about their *party choice* in the last (federal) election. Except for the United States where presidential elections are considered first‐order elections, the question relates to parliamentary elections. In contrast to many cleavage studies that group party voting into simple left–non‐left dichotomies or into party families, this study uses each party vote separately. This has the advantage of not blurring results by combining parties that may attract different electorates (e.g., combining centrist parties or various religious parties, such as Catholic versus Protestants).^4^ I restrict the number of parties to those represented to a substantial degree – that is, those with a vote share of at least 5 per cent. Respondents who voted for another smaller party/presidential candidate, for single candidates only (Switzerland), or who did not vote at all, are excluded from the analyses regarding party choice. Appendix Table 2 presents an overview of the included parties.

The second dependent variable is *electoral participation*. The measure is straightforward in asking respondents if they participated in the last (federal) election or not.

### Independent variables

As explanatory variables, I use common measures for religion and social class. These measures slightly differ between countries, but still allow for a cross‐country analysis. In terms of *religion*, whereas historically the more important conflict was between Catholics and Protestants and thus denominational, in the last few decades the difference between religious and non‐religious people became more influential in the sense of a religious–secular divide (e.g., Brooks et al. 2006; Elff 2007). I thus follow common practice in the literature and combine the two measures of denomination and church attendance (e.g., Dalton 2002; Lachat 2011). First, I split the electorate into two main denominations: Catholics versus Protestants/Anglicans. These denominations I separate further by their active religious involvement in terms of whether respondents attend church services on a regular basis or not. As a cut‐off point, I use an indicated church attendance of ‘seldom’, ‘rarely’ or ‘never’ (or respective number of attendances per year) to stand for non‐active respondents. Higher categories/values stand for active respondents. The resulting four groups are completed with a final group comprising all respondents belonging to another smaller or no denomination.^5^ Unfortunately, in Great Britain and Switzerland, the religious measure is missing in three and two elections, respectively.

From a historical perspective, *social class* has been measured differently in each country. Only recently did the International Standard Classification of Occupations (ISCO) codings allow for comparable class schemes. Hence, I use different schemes per country, which however remain stable over time, albeit with some missing data. For studying longitudinal effects and disentangling structural and behavioural effects, it is more important to have a coherent class scheme throughout all election years and less so across countries. The use of different country schemes has the further advantage of capturing particularities in the social structures of each country that may be relevant for the reasons of dealignment. For Great Britain, I use the so‐called ‘Registrar‐General's Social Classes’ (SC), comprising six classes. For the Netherlands, I use a slightly adapted version of the Goldthorpe/Erikson/Portocarero (EGP) scheme comprising seven classes. For Switzerland, I use the so‐called ‘Oesch scheme’ (Oesch 2006), comprising eight classes. For the United States, I use an adapted version of the pre‐coded occupation scheme (by the National Election Studies) following the census occupation codes comprising five classes. Appendix Table 3 presents the exact categories of the class schemes and also the missing election years.^6^

### Control variables

As control variables, I include sex, age and education. Sex is a dummy variable and age is a continuous variable. The original variables measuring education are recoded into three levels: low, middle and high. Due to data availability, for Great Britain (except for 2015) and the Netherlands I use the age when people have left school as the basis, and in Switzerland, the United States and Great Britain in 2015 I use the final degree people received. Education is a strong determinant of vertical class differences – that is, certain occupations require a certain education. Class effects may thus be underestimated when controlling for education. However, to detect class effects for horizontally equal classes (occupations) and in order to generally measure net class effects, a control for education is crucial. Besides the more conservative models including education in the main text, all models examining class effects are also calculated without education as robustness checks (gross effects). Findings of these checks are mentioned in the text (related figures can be found in the Online Appendix).

## Comparative cleavage strength

The literature offers a variety of measures for cleavage strength.^7^ Following improved technical possibilities over time, the measures have evolved significantly. A common problem of both modern and early measures, however, is the lack of consideration of changes in the size of a social group and/or party. The measure used in the following analysis solves this problem since it allows a separation of the effects due to behavioural changes and structural changes, the so‐called ‘lambda index’. This index is a modified version of the kappa index, which measures differences in voting behaviour between social groups. Originally, the kappa index was designed for binary dependent variables (see Hout et al. 1995). Lachat (2007a,b) extended it to a multinomial setting with more than two parties. In addition, the lambda index takes into account the size of the corresponding groups and parties. This is crucial for a distinction between behavioural and structural dealignment.

The first step in calculating the lambda index is a multinomial logistic regression model with party choice as dependent variable. Independent variables are the two cleavage measures of religion and social class (added separately) plus control variables. Based on the regression coefficients, predicted probabilities of party choice for each religious and class category are estimated.^8^ In a multinomial setting, the absolute lambda index looks as follows:

(1) $$\lambda_{\mathrm{absolute}}=\sqrt{\sum_{j=1}^{J}\sum_{s=1}^{S}\omega_{j}\omega_{s}{\left(\pi_{s}^{j}-{\bar{\pi }}_{s}^{j}\right)}^{2}}$$

with *j* representing the parties and *s* being the social groups (e.g., different social class categories). The probability that a member of social group *s* votes party *j* is represented by $\pi_{s}^{j}$ and the average of these voting probabilities ${\bar{\pi }}_{s}^{j}$ is defined as $\sum_{s=1}^{s}\omega_{s\;}\pi_{s}^{j}$. The ω_s_ represents the proportion of voters belonging to group *s* and ω_j_ represents the estimated vote share of party *j*.^9^ In other words, the lambda index summarises weighted deviations from the average distribution of votes per group and party (Goldberg & Sciarini 2014: 579). The resulting index ranges from 0 to 0.5, with higher values standing for a stronger impact of the cleavage (e.g., if each social group homogeneously votes for its ‘own’ party).

The main advantage of the lambda index for the present purpose is the aforementioned distinction between structural and behavioural dealignment. To do so, one weighs the calculated voting probabilities ($\pi_{s}^{j}-\;{\bar{\pi }}_{s}^{j})$ with a stable distribution in the structure of the socials groups (ω_s_). By keeping the size of the corresponding social groups constant, the resulting lambda values represent only changes in terms of behavioural dealignment. The average social group sizes of the first two election years serve as the reference category. I use the average of the first two years to have a more solid and reliable basis and to accommodate for possible particularities of the earliest data point used.

For the analysis of political dealignment and related turnout gaps, I run logistic regression models with electoral participation as dependent variable (dummy). Again, the religious and social class variables, plus the basic controls, serve as independent variables. Based on the regression coefficients, I calculate predicted probabilities of participation for all cleavage groups. The calculation includes a correction for the general overestimation of turnout in surveys for each election year. As the main interests are relative differences between certain categories over time, a correction of the turnout bias is necessary to derive valid results.^10^

## Results

### Religious cleavage voting (structural versus behavioural dealignment)

**Figure 1:**
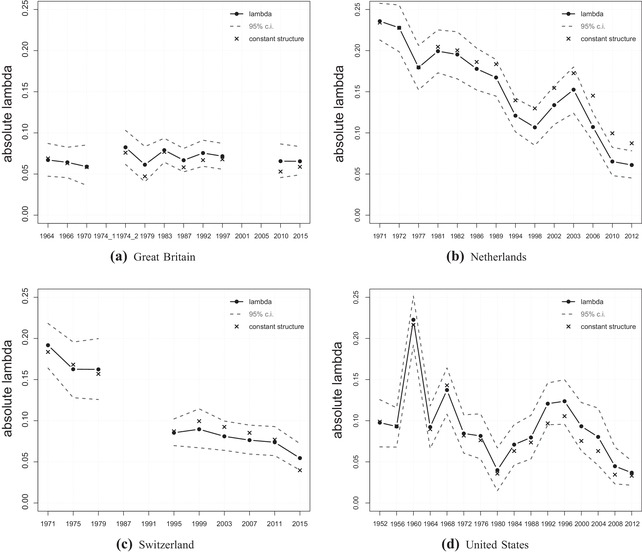
Lambda index for religion.

The results start with the religious cleavage and its separation into structural and behavioural changes. Figure 1 shows the developments for all four countries. Each country plot includes the overall development of cleavage strength (solid black line) with 95 per cent confidence intervals (dashed grey lines) and the development with an imaginary stable social structure (x‐marks). The y‐axis represents the lambda values – that is, how homogeneously each cleavage group votes for ‘their’ party – and the x‐axis shows the election years. A reading example to separate the effects due to behavioural or structural changes in the graphs is the following: the evolution of the normal lambda values includes both structural and behavioural effects. Regarding only the values for the constant social structure (x‐marks) displays changes based solely on the behaviour of social groups. Since the group size is fixed and the same as in the earliest two elections, all changes in lambdas are due to the groups’ behaviour. The comparison between the values based on the actual structure and the constant one in each single election year displays structural changes. Whenever the values of the constant structure (x‐marks) are above the normal values (circles), this stands for structural dealignment compared to the first two election years. The development of these gaps between normal and constant values in later elections thus provides a comparison of to what extent structural changes influenced the cleavage strength between certain elections (growing gaps stand for ongoing impact of structural changes).

The development of Swiss religious voting is the easiest to interpret (c). The lambda index shows a clear decline between the 1970s until the 1990s, which afterwards flattens out, but the decline still continues during the last elections. The values holding the social structure constant run almost parallel to the normal lambda line, meaning that in Switzerland the decrease in religious voting is not due to structural changes, but to changes in the behaviour of the religious groups.^11^ The overall decline of religious voting is also present in the Netherlands (b) and even slightly more pronounced – however, the underlying reasons differ. Comparing the solid line of the lambda measure with the x‐marks representing the constant structure shows that although both patterns significantly decrease over time, the decrease using the constant structure is less steep, especially since the elections of 2006.^12^ Due to the still substantial decline of the constant lambda values, the overall decline is again mainly a result of behavioural changes, but this time the change of the religious structure added to the overall decline. With an unchanged religious structure, the cleavage would be more important nowadays.

The pattern in Great Britain (a) does not show an overall decline. The values in the 1960s are already on a low level and remain there with only minor variations over time. In the absence of an overall decline, the values of the constant structure are unsurprisingly also very stable over time. Finally, the pattern for the United States (d) is the most volatile. A clear decline is not observable as the values in the 1950s are already quite low. The elections in 1960 represent an outlier with a very strong influence of religion on voting behaviour. Although the recent decline since 1996 indicates a very small influence of religion in the United States nowadays, the overall rather volatile pattern does not allow dismissal of religion in future elections. Given that the values of the constant structure are similar to the normal values, the changes are again mainly due to behavioural effects instead of structural ones. On a side note, the religious pattern in the United States is a nice example of how decisive the time horizon is for an analysis of cleavage influence. Without the comparatively low cleavage impact in the 1950s, the pattern would look much more like a clear decline and less like a volatile fluctuation over time.

In sum, the patterns of the four countries are quite different. First, the overall development of the religious cleavage differs between most countries, only the Netherlands and Switzerland show a clear decline. Second, in case of a similar decrease of religious voting in these two countries, the underlying reasons are slightly different between behavioural effects only (Switzerland) and a little add on of structural effects to behavioural effects (the Netherlands).

### Social class cleavage voting (structural versus behavioural dealignment)

**Figure 2:**
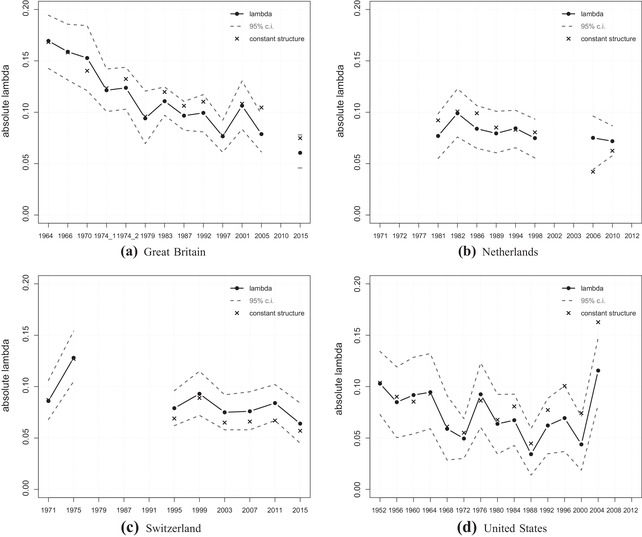
Lambda index for social class.

The diverse pattern across countries is also observable for the social class cleavage (Figure 2). This time, the decreasing trend in Great Britain (a) is the clearest. Since the 1960s, class voting lost almost linearly in importance. The pattern is slightly more stable between the late 1970s until the early 2000s, but especially in the most recent elections the importance of class voting is fairly low.^13^ The values with a constant social structure are almost always above the actual lambda values, but still clearly inside the confidence intervals. This changed in the latest two elections, so that, recently, structural effects are getting more important and have partly contributed to the significant decline of class voting since 2001. The main part of the decline, though, is due to behavioural changes.

In the Netherlands (b), class voting was already on a low level in the 1980s (no earlier data available) and remained on that level in the last decades. Interesting are the values of the constant structure in 2006 and 2010, which are below the actual lambda values. This means that social groups that have grown larger over time – for example, higher classes such as managers – must have become more polarised than shrinking groups like manual workers. Without these structural changes, the class effect would be even weaker than it is already.

A similar pattern is observable in Switzerland (c). Here, the values with a constant social structure are also below the actual lambda values, though again still within the confidence intervals. Structural changes may have partly contributed to dampen the decline in class voting, but the observable decline since 1975 is mainly due to behavioural changes.

Finally, the pattern in the United States (d) is again a trendless fluctuation over time and interestingly on a similar level as the other three countries. Although class impact for party choice is said to be comparatively low in the United States, nowadays it is not lower but higher than the other three countries. Until the mid‐1990s, the values of the constant structure do not show any particular pattern. However, since then, the values are clearly above the normal lambda standing for structural dealignment – that is, some (growing) social classes are becoming less homogeneous in their voting behaviour. Without that structural dealignment (regarding only the constant lambdas) the recent slight upwards trend would be even stronger. Whether this positive trend continues remains to be seen given the strong fluctuations over time. The robustness checks excluding education as a control variable (gross effects) confirm the discussed findings (see Appendix Figure 2).

### Political dealignment among social classes

**Figure 3:**
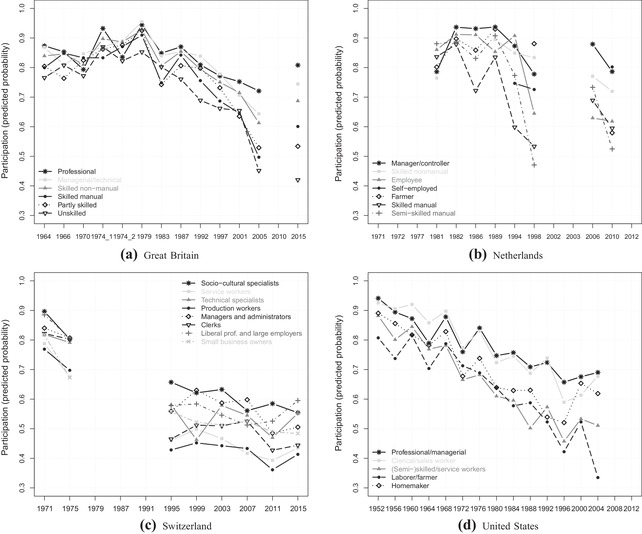
Turnout across social classes.

Having examined behavioural and structural dealignment in cleavage voting, I now turn to political dealignment – that is, the abstention of specific cleavage groups. Figure 3 displays predicted probabilities of turnout among all social class categories over time (based on binary logistic regression models). A commonality between the patterns in all countries is the higher heterogeneity in turnout between classes in recent elections. In the first elections under study, differences in turnout are only around 10–15 percentage points between the highest and lowest levels of turnout. Especially in the context of overall high levels of turnout, these differences are relatively small. In contrast, turnout in recent elections shows differences of around 40 percentage points in Great Britain and the United States. In the Netherlands and Switzerland, the maximal differences in turnout are smaller (30 percentage points in the former and 20 in the latter), but have still increased significantly over time. Thus, although in general turnout has declined among all classes, the speed and magnitude of that decline differs significantly between classes. There would be several interesting aspects to discuss for specific classes in some of the countries. For the analysis of a growing turnout gap, though, I examine the two extreme categories per country in more detail.

**Figure 4:**
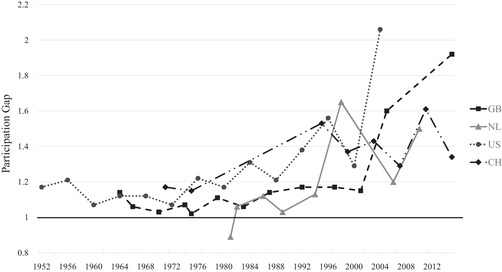
Class gap in participation over time. Notes: Great Britain (GB), the Netherlands (NL), Switzerland (CH) and the United States (US). The gap in turnout (participation ratio) is calculated by dividing the predicted probability of a (or the most) strongly participating class versus a little (or the least) participating class. In GB these are professionals versus unskilled, in NL managers/controllers versus semi‐ or unskilled manual, in CH sociocultural specialists versus production workers and in the US professionals/managers versus labourer/farmer. The black horizontal line at 1 stands for an equal participation between both class categories.

In order to better grasp the effect of political dealignment on a turnout gap, particularly as absolute turnout differences are not straightforward to interpret in a context of overall declining participation, Figure 4 presents the relative differences in turnout between the most and least participating classes (i.e., lower and higher social classes) over time in a graphical way (the exact numerical values are displayed in Appendix Table 4). Values represent the participation ratio between both classes. A value of one (black horizontal line) represents a perfectly equal participation between the most and least participating classes and values above one and growing stand for an increasing political dealignment of the lower class compared to the higher one. The development nicely shows how much differences in turnout have grown over time.

Great Britain has witnessed the most extreme change. After a rather equal participation until 2001, a large gap emerged resulting in a turnout for the higher classes (professionals) around twice as high as for the lower classes (unskilled) in 2015. The 2015 gap may be an exception, especially given the – against the common trend – increasing participation of the higher classes shown in Figure 3a. However, since the widening of the participation gap already began in the 2005 elections, this hints at growing differences in turnout. In the Netherlands, turnout was also rather equal until the 1990s before a significant participation gap evolved as well. It is less extreme compared to Great Britain and also more volatile, but the more well‐off classes (managers/controllers) still participated around 1.5 times as much as the less well‐off classes (semi‐ or unskilled manuals) in 2010. The Swiss time‐series is very similar to the Dutch one, showing substantial differences already in the mid‐1990s with fluctuations thereafter, but always a larger turnout gap than in the 1970s. Finally, the pattern in the United States shows a more gradual increase of the turnout gap starting comparatively soon in the late 1970s/early 1980s and with a significant gap emerging in the more recent elections in 2004. In that year, the higher classes (professionals/managers) participate around twice as much as lower classes (labourers/farmers), resulting in an even slightly larger gap than in Great Britain in 2015. This confirms the earlier discussed importance of a class bias in turnout in the United States. However, as the results show, this is not (any more) a specific characteristic of the United States, but to a different degree political dealignment across classes is present in all four countries under study. The related increasing class division on turnout in all countries confirms *H3*. Overall, the development looks like a more modern phenomenon happening particularly from the 1990s onwards, so that political dealignment and its effects across classes may become even more pronounced in the future. The robustness checks excluding education as a control variable (gross effects) again confirm the findings (see Appendix Figures 3 and 4). Interesting for the case of the United States is that controlling for education weakens an even stronger class bias in turnout in the earlier years, but not in the more recent years. This means that education lost its explanatory power for class differences in turnout.

### Political dealignment among religious groups

**Figure 5:**
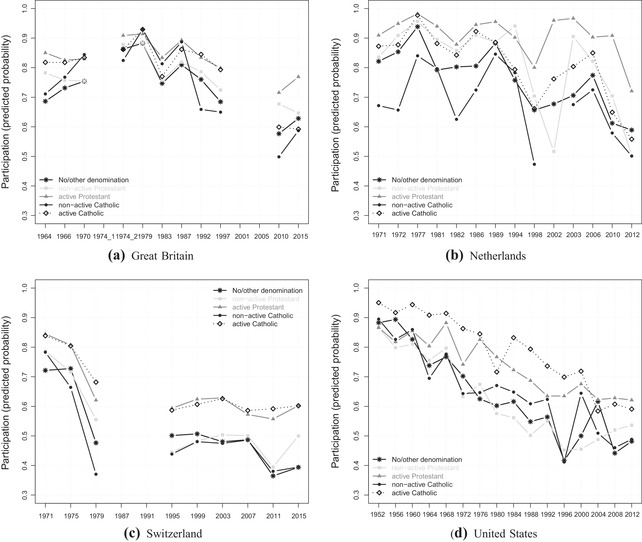
Turnout across religious groups.

In order to see whether political dealignment is a specific phenomenon for the social class cleavage, I now turn to the same analysis regarding religion. Figure 5 displays the predicted probabilities of turnout among the five religious groups. Although the trend is somewhat less clear than for the class cleavage, we can again see an increasing heterogeneity across religious groups in the context of a general decreasing participation. Particularly in Switzerland and the United States, and to some extent also in Great Britain, the turnout levels of the religious categories were much more similar in the first elections under study. Over time, the turnout gaps have widened. It is interesting that in both Switzerland and the United States, the divide (today) is clearly between religious and non‐religious people and not between denominations. Both active categories of Protestants and Catholics show similarly high participation levels in contrast to much lower levels for the remaining three groups comprising the non‐active denominational groups and mainly people without any denomination. Belonging to a church – at least on paper – without being actively involved does not result in a high turnout. Furthermore, in both countries the absolute differences between groups are larger in the 1970s and 1980s, decreasing until the more recent elections. The Dutch case with its volatile pattern over time and across religious groups does not really fit with the other three countries. For a simpler interpretation of a potentially widening turnout gap, I again focus on the extreme categories in more detail.

**Figure 6:**
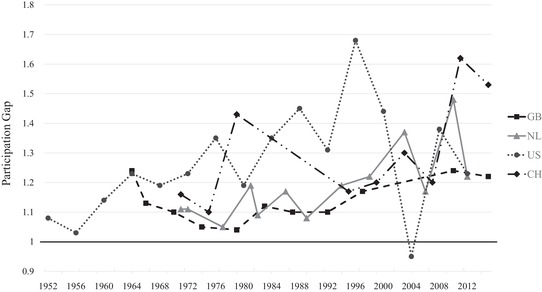
Religious gap in participation over time. Notes: Great Britain (GB), the Netherlands (NL), Switzerland (CH) and the United States (US). The gap in turnout (participation ratio) is calculated by dividing the predicted probability of the most strongly participating religious groups versus the group comprising people without or with another denomination. In GB and NL the highest participating groups are active Protestants, and in CH and US these are active Catholics. The black horizontal line at 1 stands for an equal participation between both religious groups. Please also note the different y‐axis scale compared to Figure 4.

Figure 6 displays the gap between active Protestants (Great Britain and the Netherlands) or active Catholics (Switzerland and the United States) versus people with another or no denomination (exact numerical values displayed in Appendix Table 5). The latter category is used to enable a better comparison between the four countries, although this category is not in all four countries the least participating group. Overall, we again observe growing turnout gaps, but with different magnitudes and more volatility compared to the class gaps. For instance, both the United States and Switzerland show increasing turnout gaps, but the pattern in the United States especially is volatile with the biggest differences already present in the late 1980s and 1990s. In Switzerland, the latest two elections show a particularly big turnout gap of participation by active Catholics that is more than 1.5 times as high as the one by people not belonging to the Church. The Netherlands also displays an increasing pattern, but on an overall lower level and with strong volatility in the latest elections. The most stable pattern exists in Great Britain, where the current gap is almost the same as already in the 1960s. The, on average, rather small gaps tally with the minor role religion plays in British politics (‘secular world’). Compared to the 1970s and 1980s, though, the pattern also shows a steady increase in the turnout gap.^14^ Although the gaps are not as large as for the class cleavage and neither increase as linearly and strongly, political dealignment is also present for the religious cleavage and leads to a vote‐abstention division across religious groups as well, which thus confirms *H4*. As theorised earlier, the stronger civic skills of religious people may lead them to resist the general trend of abstention more strongly, and this especially in contexts with a still ongoing relevance of religious topics on the political stage – for example, in terms of morality.

To sum up all three forms of dealignment, the strongest similarity across countries concerns the importance of political dealignment and related growing differences in electoral participation. These differences are observable for both the religious and social class cleavage (confirming *H3* and *H4*), though in a clearer and stronger way for social class. Especially the more recent elections display (very) large turnout gaps between lower and higher classes. Furthermore, political dealignment evolves independently of traditional cleavage voting. Political dealignment appears around the same time in all four countries, whereas the time of the decline in cleavage voting strongly differs between countries. The ‘very strong relationship’ between declining cleavage voting and an increasing voting versus abstention division for class in Great Britain by Heath (2018: 1062) can thus only partly be confirmed. Although my results display a similar pattern for British class voting, albeit much weaker than in Heath (2018), the results do not confirm such a systematic relationship across countries and neither for the religious cleavage. A confirmation of *H1* is that if a decline in cleavage voting is present – either regarding religion or social class – behavioural changes matter. The importance of structural effects, in contrast, is less clear. Partly, structural changes in the society add to the decline of cleavage influence fostered by behavioural changes, such as religious voting in the Netherlands or class voting in Great Britain and the United States. In other cases, though, structural effects do not matter at all (e.g., religious voting in Switzerland) or they even show reverse effects that helped to maintain a certain level of cleavage influence (e.g., class voting in the Netherlands and Switzerland). As a result, *H2* is not confirmed.

## Conclusion

The goal of this article was to analyse the underlying mechanisms of declining cleavage voting in four Western countries. Alongside structural and behavioural effects that relate to changes in party choice, the article expanded the concept of cleavage voting with a turnout perspective by also examining political dealignment. This is to argue that class and religious groups not only differ in their party choice, but increasingly also in their decision to vote at all. The results confirm this argument by showing that certain groups exhibit a steeper participation decline than other groups, which results in growing differences in turnout. These differences are particularly large in the class cleavage where lower classes such as (unskilled) workers decide increasingly more often to abstain compared to higher classes such as managers or professionals. This gap especially developed in the more recent elections, meaning that this misrepresentation of social groups among the politically active part of the population may worsen in the future. The phenomenon, though, is not a unique pattern among social classes. As the results show increasing differences in turnout across religious groups as well, religiously active people also become increasingly over‐represented among the politically active population.

Besides highlighting the importance of including electoral participation in cleavage studies, the article also provides clear answers about the underlying reasons for the commonly found decline in cleavage voting. Such a decline is almost entirely due to behavioural changes among social groups (i.e., party–voter links significantly weakened) and is not due to changing group sizes (i.e., former important groups such as workers or religious people declined in size). Regarding the ongoing discussion about the extent of the decline in cleavage influence, the four countries confirm a significant decline – except the decline had occurred already before the time period under study here, which is the case for both the class and religious cleavage in the United States. It is interesting that class voting in the United States, which is commonly found less important, lately shows increasing influence. Further noteworthy about the timely occurrence of the three mechanisms is that the decline in traditional cleavage voting is not directly linked to the increase in political dealignment. Although the timing and strength of behavioural dealignment differs between the countries under study, political dealignment occurred much more in parallel across all four countries. However, political dealignment might require weak cleavage voting in the first place. In that sense, people first lose their traditional party representative and afterwards lose contact with politics in general. The study of the precise link between these two processes and the exploration of the exact reason for the parallel occurrence of political dealignment across countries is something for future research.

A limitation of this research is its exhaustive focus on the demand side of cleavage voting, though due to space limitations it has not been feasible to expand the investigation. Similar to Evans and De Graaf's (2013) study that analyses the supply side of traditional cleavage voting, analyses of the supply side of political dealignment are needed (see Heath (2018) for first results). Such analyses could answer the question of the role of individual parties, but also the role the political system as a whole plays in the increasing abstention of certain groups from political life. However, to continue the examination of the strong diverging trends in political dealignment this study has found is already of utmost importance. In case the trends continue with similarly dramatic changes, such as the class gaps in the United States and Great Britain, this may eventually endanger the political system since significant parts of the electorate will be (self‐)excluded.

## Supporting information


**Table 1: List of data from national election studies**
**Table 2: Parties included per country and year**
**Table 3: Social class schemes**
**Table 4: Participation gap between most and least participating classes**
**Table 5: Participation gap between active Catholics/Protestants and people with no or another denomination**

**Appendix Figure 1: Religious gap in participation over time, alternative calculation**.

**Appendix Figure 2: Lambda index for social class (without education as control)**.

**Appendix Figure 3: Turnout across social classes (without education as control)**.

**Appendix Figure 4: Class gap in participation over time (without education as control)**.

## Notes

1 Although behavioural dealignment most often refers to a proactive change of the electorate's preferences, it may also be a reaction to changing party strategies. Ladner (2004: 308) argues that it is possible that parties nowadays mobilise less around former sociostructural differences, which results in changing voting behaviour among former party supporters. Additionally, parties might get more votes from previous opposing social groups. In sum, such a process fuelled by changing parties leads to the same weakening of previously strong ties between parties and social groups. For a recent study focusing on the supply side, see Evans and De Graaf (2013).

2 The mentioned developments are especially harmful for parties representing traditional cleavages. However, the changes are not a unique problem for those parties, as the discussed developments also influence the support for (more modern) parties representing other/new ideologies. As Clark et al. (1993) argue, parties in general suffered by increasing levels of education, mass media and growing strength of issue‐specific groups.

3 See, e.g., Evans's (2000: 407) criticism of data simplifications in comparative studies:
Apart from its simplicity, the main reason for the use of a dichotomy to represent voter choice seems to have been a desire to make systematic cross‐national and over‐time comparisons. Unfortunately, the selective nature of what is being compared undermines any true comparability. The problem is analogous to that faced in the analysis of class position, in that changes in the composition of composite categories such as left or nonleft may lead to spurious changes in estimates of class voting.

4 It further avoids uncertainties in the interpretation of results that might be purely due to recoding decisions, such as ‘to collapse the party choice variable into left versus right, thus ignoring the complexity of the “new left” and the “new right”, for example’ (Evans & De Graaf 2013: 392).

5 Since people belonging to another denomination is a very small group, it is not possible to include them as a separate category, although it theoretically could make sense (e.g., the high turnout among Jews in the United States). As some datasets like the American National Election Study (ANES) already grouped smaller denominations with no‐denominations, I decided to follow this coding, which for most other denominations makes sense. For example, Muslims do not have a specific representative in the party system compared to Christian denominations.

6 Unfortunately, there are more missing years for social class than already present for the religious variables. In some cases, one could use a different or less detailed class scheme instead. However, such an approach would hinder a coherent longitudinal analysis, so that one could not know if found effects are due to actual changes over time or rather to a different class coding in a given year (e.g., Evans 2000). For this reason, I decided against the use of alternative class measures in the election years with missing data.

7 For a detailed overview of available measures, see Lachat (2007a,b) and Hug and Trechsel (2002).

8 For the calculation I use the Stata program *cindex*, written by Romain Lachat (http://www.romain-lachat.ch/software.html). As the data from GB and CH include sample weights due to an over‐representation of certain regions and cantons, in these two cases I use *cindexw*, which incorporates the option of weighting. The control variables are fixed at the following values to calculate predicted probabilities: gender = male, age = 45 years and education = middle.

9 For both the calculation of the predicted probabilities for a certain party choice and the subsequent computation of confidence intervals, the used program *cindexw* relies on simulation techniques. First, the program simulates the distribution of the regression parameters after each model estimation. Based on these simulated parameters it computes a predicted value together with the corresponding cleavage strengths (lambda index). The retrieved information about the distribution of these indices then allows for calculation of their average value and confidence interval (Lachat 2007b).

10 Theoretically, turnout bias consists of over‐representation of voters (selection bias) and over‐reporting by respondents (misreporting bias) (e.g., Sciarini & Goldberg 2017; Goldberg & Sciarini 2019). Whereas misreporting should be a rather stable phenomenon, the selection bias is more important for a longitudinal analysis. In times of decreasing survey response rates, selection bias might result in growing over‐representation of voters in more recent surveys and a subsequent stronger over‐estimation of turnout.

11 As the missing religious data (due to missing church attendance) leads to a comparatively long gap between 1979 and 1995, I ran another model using three groups of (no) denomination. This model confirms the significant drop between the 1970s and 1990s and the found importance of behavioural, not structural changes (see also Goldberg 2017).

12 The constant values are even outside the confidence intervals of the normal lambda pointing to a significantly bigger gap in the latest three elections. However, note that for reasons of readability the confidence intervals of the values using the constant structure are not displayed. Hence, in a strict sense one cannot speak of statistically significant differences here.

13 The different patterns of the religious and class cleavage in Great Britain with an already occurred decline of religious voting before the first election under study and much later following the class cleavage decline is in line with the results by Franklin et al. (1992).

14 For the Netherlands and Great Britain, I also calculated the gap compared to non‐active Catholics, as in most election years this group is the least participating one in both countries. Appendix Figure 1 shows the respective turnout patterns. For Great Britain, an increasing turnout gap is now more clearly observable; for the Netherlands the pattern shows again significant turnout gaps, but without a clear increase due to even stronger volatility over time.
