# Supplementary material for: The evolution of cleavage voting in four Western countries: Structural, behavioural or political dealignment?
Source: Eur J Polit Res. 2019 Mar 29;59(1):68–90. doi: 10.1111/1475-6765.12336 (PMC7003807; doi:10.1111/1475-6765.12336)
Supplement: Supplementary file 1 — Table 1: List of data from national election studies Table 2: Parties included per country and year Table 3: Social class schemes Table 4: Participation gap between most and least participating classes Table 5: Participation gap between active Catholics/Protestants and people with no or another denomination [file EJPR-59-68-s005.doc]

Appendix


Table 1: List of data from national election studies


Country	Year(s)	No. of cases	Name	
				
GB	1964, 1966, 1970	2922	1963–1970 Political Change in Britain	
GB	1974 (1)	2462	1974 February BES Cross-Section	
GB	1974 (2)	1178	1974 October BES Cross-Section	
GB	1979	1893	1979 BES Cross-Section	
GB	1983	3955	1983 BES Cross-Section	
GB	1987	3414	1987 BES Cross-Section	
GB	1992	3534	1992 BES Cross-Section	
GB	1997	3615	1997 BES Cross-Section	
GB	2001	3035	2001 BES Cross-Section	
GB	2005	4791	2005 BES Post Election Survey	
GB	2010	3512	2010 BES Cross-Section	
GB	2015	2987	V 3.0. 2015 Face-to-face Post-election Survey	
see http://www.britishelectionstudy.com/data-objects/cross-sectional-data/	
				
  NL	1971, 1972, 1977,	  22821	  DPES integrated file 1970–2006	
	1981, 1982, 1986,			
	1989, 1994, 1998,			
	2002, 2003, 2006			
NL	2010	2621	DPES 2010	
NL	2012	1677	DPES 2012	
see http://www.dpes.nl/en/ 	
				
  CH	1971, 1975, 1979,	  31668	  Selects Cumulative File 1971–2011	
	1987, 1991, 1995,			
	1999, 2003, 2007,			
	2011			
CH	2015	5337	Swiss Electoral Studies (Selects) 2015	
see http://forscenter.ch/de/our-surveys/selects/

US	1952–2012	55674	ANES Time Series Cumulative Data File 
see http://www.electionstudies.org

Table 2: Parties included per country and year

Country	Year(s)	Parties	

GB	
1964–1987	
Conserva	
GB	1964–1987	Conservatives, Labour, Liberal	
GB	1992–2010	Conservatives, Labour, Liberal, SNP	
GB	2015	Conservatives, Labour, Liberal, SNP, UKIP	

NL	
1971–1986	
PvdA, CDA, VVD, D66	
NL	1971–1986	PvdA, CDA, VVD, D66	
NL	1989–1994	PvdA, CDA, VVD, D66, GroenLinks	
NL	1998	PvdA, CDA, VVD, D66, GroenLinks, SP	
NL	2002–2003	PvdA, CDA, VVD, D66, GroenLinks, SP, LPF	
NL	2006–2012	PvdA, CDA, VVD, D66, GroenLinks, SP, PVV	

CH	
1971–1979	
FDP, CVP, SP, SVP	
CH	1971–1979 FDP, CVP, SP, SVP	FDP, CVP, SP, SVP	
CH	1987–2015	FDP, CVP, SP, SVP, GP	
			


US	1952–2012 FDP, CVP, SP, SVP	Republicans, Democrats	
			


Note: In the Netherlands 1971 and 1972 the CDA vote is a combination of voting for the three parties KVP, ARP
and CHU. In Switzerland the FDP vote includes the LPS (until 2007) and the CVP vote the CSP.

Table 3: Social class schemes

Country	Class categories	


GB	Professional occupations
Managerial and technical occupations
Skilled non-manual occupations
Skilled manual occupations
Partly-skilled occupations
Unskilled occupations	


NL	Manager / Controller
Skilled nonmanual / Higher professional
Employee / semi-skilled nonmanual / manual supervisor
Self-employed
Farmer
Skilled manual
Semi-skilled or unskilled manual	


CH	Socio-cultural specialists
Service workers
Technical specialists
Production workers
Managers and administrators
Clerks
Liberal professions
Small business owners	


US	Professional and managerial
Clerical and sales workers
Skilled, semi-skilled and service workers
Laborers / Farmers, farm managers, farm laborers and foremen
Homemakers	

Note: Missing data for GB 2010, NL 1971-1977 and 2002/2003, CH 1979-1991, US 2008/2012

Table 4: Participation gap between most and least participating classes


GB


NL


CH


US


Note: The values are calculated by dividing the predicted probability of a (or the most) strongly participating class versus a little (or the least) participating class. A value of 1.17 in 1971 in CH thus equals a participation of socio-cultural specialists which is 1.17 times higher than the one for production workers.


Table 5: Participation gap between active Catholics/Protestants and people with no or another denomination


GB


NL


CH


US


Note: The values are calculated by dividing the predicted probability of active Catholics (CH & US) or active
Protestants (GB & NL) versus people with no or another denomination.


- Figure 8 about here -
- Figure 9 about here -
- Figure 10 about here -
