# Supplementary material for: The evolution of cleavage voting in four Western countries: Structural, behavioural or political dealignment?
Source: Eur J Polit Res. 2019 Mar 29;59(1):68–90. doi: 10.1111/1475-6765.12336 (PMC7003807; doi:10.1111/1475-6765.12336)
Supplement: Supplementary file 2 — Appendix Figure 1: Religious gap in participation over time, alternative calculation. [file EJPR-59-68-s001.pdf]

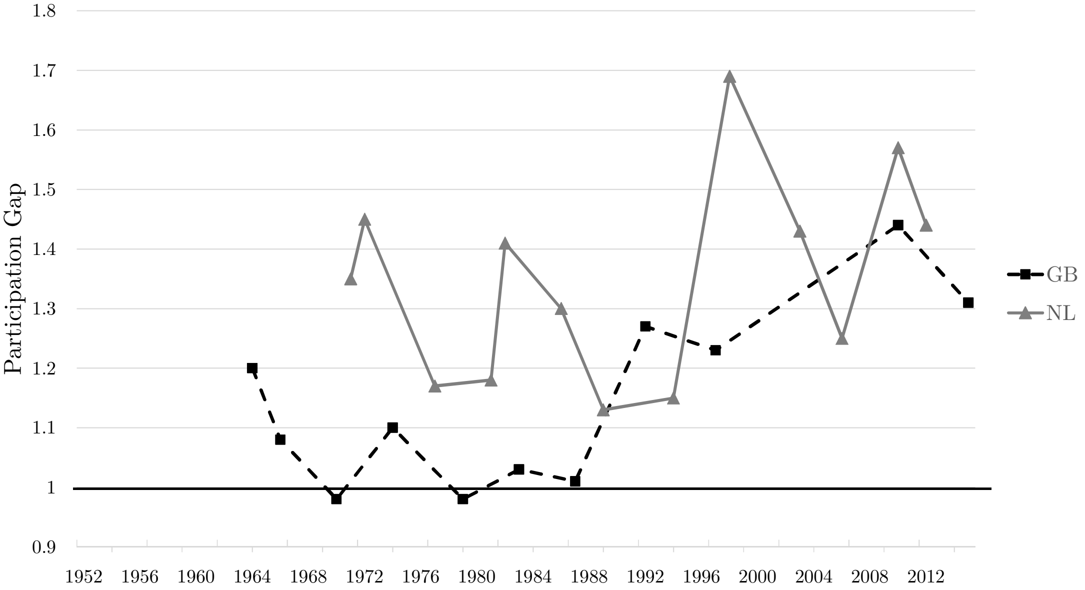

Note: The gap in turnout (participation ratio) is calculated by dividing the predicted probability of active Protestants versus non-active Catholics.
