# Supplementary figures and images for: The evolution of cleavage voting in four Western countries: Structural, behavioural or political dealignment?
Source: Eur J Polit Res. 2019 Mar 29;59(1):68–90. doi: 10.1111/1475-6765.12336 (PMC7003807; doi:10.1111/1475-6765.12336)

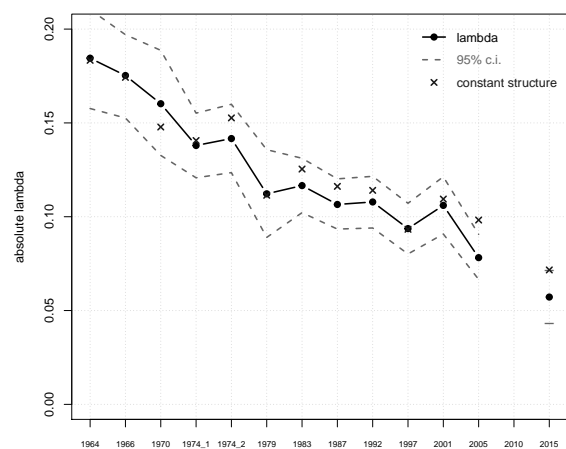

(a) Great Britain

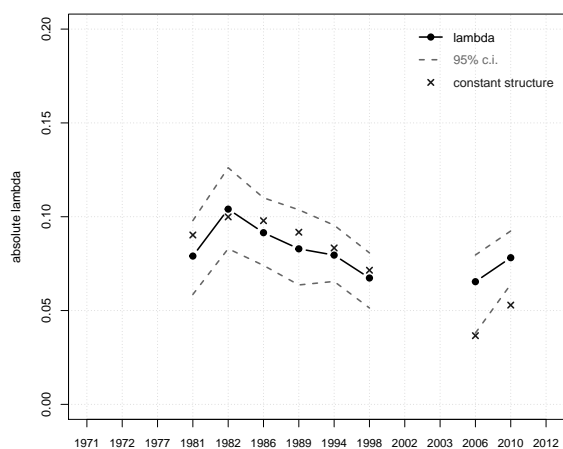

(b) Netherlands

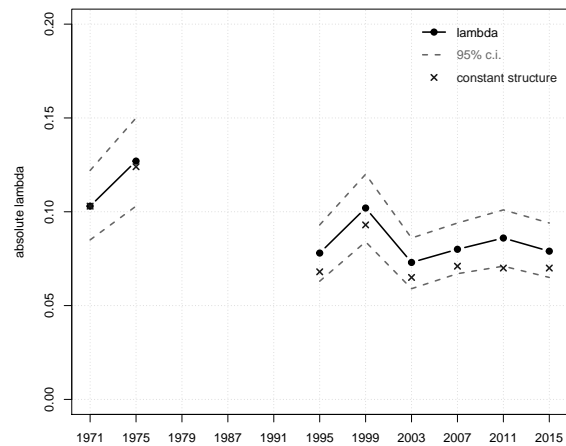

(c) Switzerland

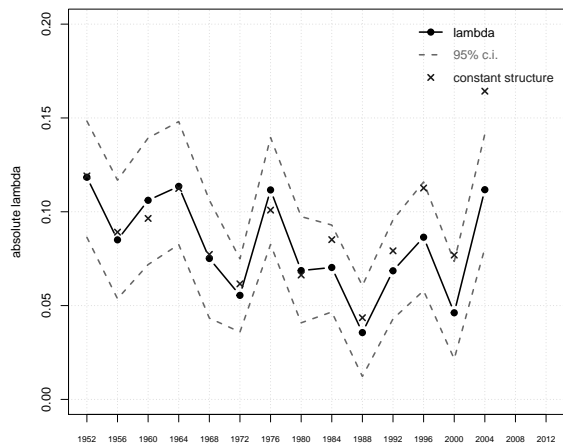

(d) United States

Supplement: Supplementary file 3 — Appendix Figure 2: Lambda index for social class (without education as control). [file EJPR-59-68-s002.pdf]

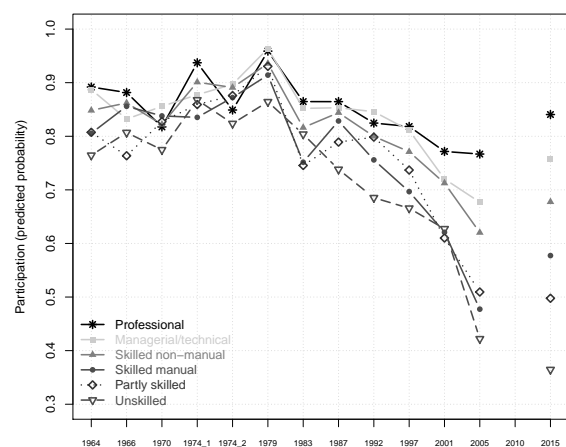

(a) Great Britain

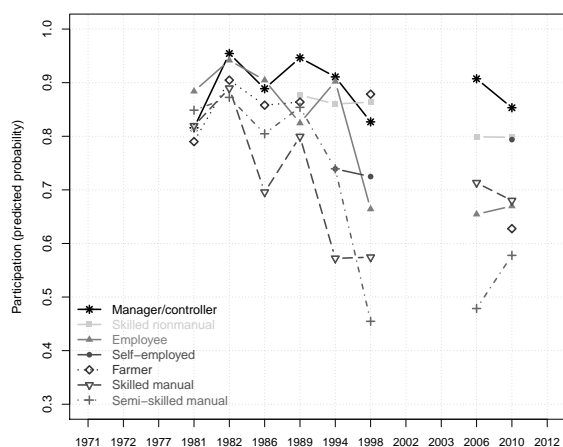

(b) Netherlands

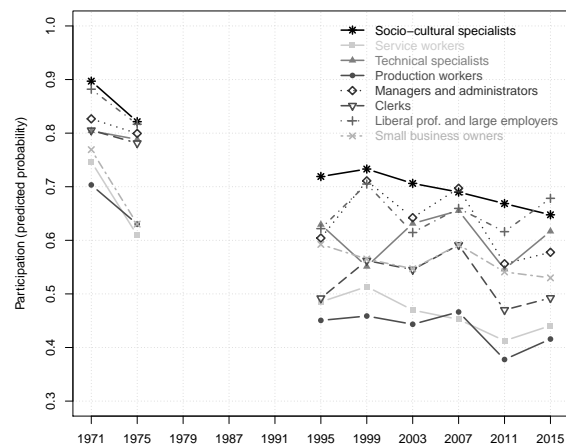

(c) Switzerland

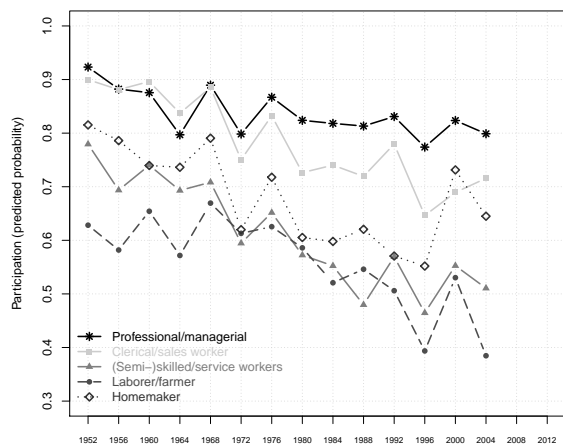

(d) United States

Supplement: Supplementary file 4 — Appendix Figure 3: Turnout across social classes (without education as control). [file EJPR-59-68-s003.pdf]

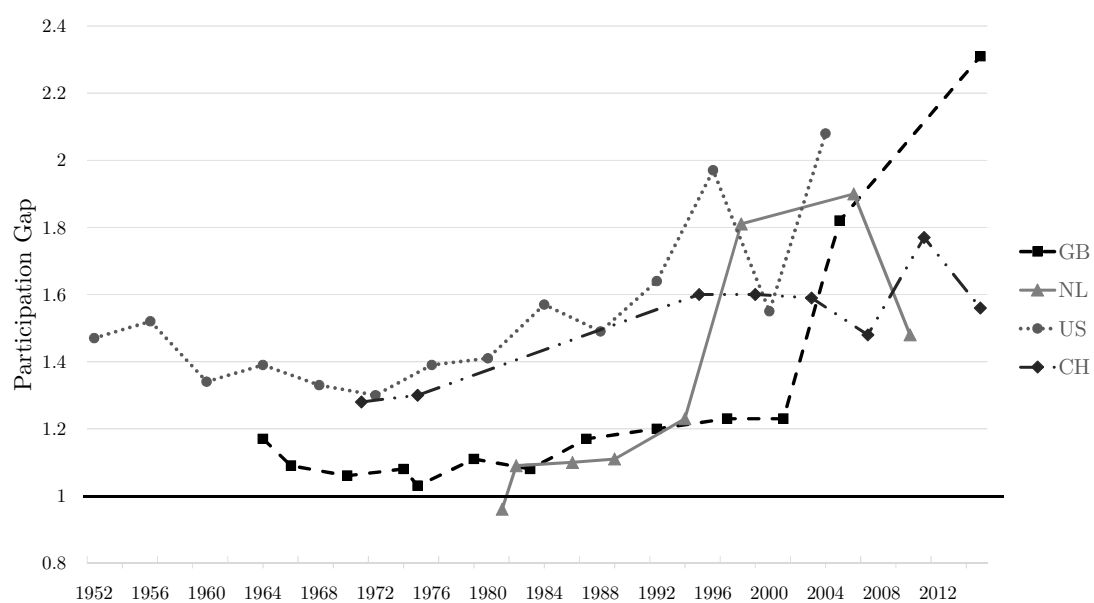

Supplement: Supplementary file 5 — Appendix Figure 4: Class gap in participation over time (without education as control). [file EJPR-59-68-s004.pdf]
